# Discerning Fact From Fiction: An Assessment of Coronavirus-19 Misinformation Among Patients in Rural Michigan

**DOI:** 10.7759/cureus.21710

**Published:** 2022-01-29

**Authors:** Vivian Wang, Samantha E Liu, Renee Fuller, Chin-I Cheng, Neli Ragina

**Affiliations:** 1 Internal Medicine/Pediatrics, Central Michigan University College of Medicine, Midland, USA; 2 Medicine, Central Michigan University College of Medicine, Mount Pleasant, USA; 3 Family Medicine, MidMichigan Health, Midland, USA; 4 Statistics, Actuarial and Data Science, Central Michigan University, Mount Pleasant, USA; 5 Genetics, Central Michigan University College of Medicine, Mount Pleasant, USA

**Keywords:** knowledge-behavior gap, patient-provider communication, rural health, misinformation, coronavirus-19

## Abstract

Coronavirus-19 misinformation poses a unique challenge for public health communication efforts. In rural communities, COVID-19 misinformation is not well studied. We investigate patients’ ability to discriminate COVID-19 fact from fiction from their news sources, as well as general COVID-19 knowledge, perceptions, public health practices, and their primary news sources in 258 adult patients at a primary health clinic in rural Michigan. Most of the population surveyed was able to correctly differentiate reliable COVID-19 public health information from fabricated information. However, only 55.4% of participants reported that they would be somewhat or extremely likely to get a COVID-19 vaccine. The most reported news source was mainstream broadcast television channels such as CBS and ABC. Our data support those older participants are better informed and more likely to practice safe public health practices than younger participants. Based on our data, we offer strategies for public health campaigns in rural communities, such as targeted interventions towards younger people and utilizing local television stations and community institutions to disseminate public health communications and health promotions. Public health interventions beyond education should be considered to mitigate the gap between COVID-19 knowledge and prevention behaviors. Future studies should investigate the role of health care providers in COVID-19 communication with patients, understanding hesitations toward COVID-19 vaccination, and communication strategies to best increase COVID-19 vaccine uptake in rural communities.

## Introduction

Implementing effective public health communication strategies to combat COVID-19, a disease caused by SARS-CoV-2, continues to be a challenge in the United States and worldwide. Since the beginning of its spread in the USA, there have been rapidly evolving data on the virus. Media stories and safety guidelines quickly became outdated and updated, resulting in public confusion and mistrust [1,2], thus making public health interventions difficult to implement. Problems interpreting fact from fiction in the media may potentially have devastating effects. Thus, competency in deciphering misinformation in the media is crucial in keeping communities safe during these times [3].

Misinformation is characterized as wrong information without intent to be deliberately misleading [4]. Prominent predictors of belief in misinformation include lower trust in science, scientists, journalists, and mainstream media [5]. The novelty of COVID-19 coupled with a universal lack of information about the virus generated an environment that allowed misinformation to thrive. Social media then allowed for quick dissemination of both truthful and untruthful COVID-19 information.

Public health crisis response activities on the ground and online are becoming increasingly simultaneous and intertwined. Misinformation can weaken public trust in research and hinder public health efforts, such as social distancing or vaccination, in battling COVID-19 [6]. There is also a need for a more proactive and agile public health presence in media to combat the spread of “fake news,” in particular in combination with infectious disease outbreaks. Social media can be used in positive ways to disseminate public health knowledge and information directly to the public. However, media can also be a powerful weapon and, if not used appropriately, can be destructive to public health efforts, especially during a public health crisis. Understanding where the public gets their information regarding COVID-19 is important to inform epidemiological efforts to manage the COVID-19 pandemic.

Research on the harmful effects of misinformation in the fight against COVID-19 is growing. However, rural representation in COVID-19 communication and misinformation studies is lacking. Rural communities are more vulnerable to COVID-19 for a number of reasons, including fewer physicians, access to care, and a greater burden of social determinants of health [7]. Social vulnerabilities, such as living in a rural area, have been found to be associated with lower health literacy [8]. Coupled with how rural populations are on average older than non-rural populations, this demographic may especially be susceptible to misinformation [9,10]. Additionally, rural residences may favor and trust certain media sources over others [11]. The effects of COVID-19 misinformation and poor health communication on rural health systems are not well studied. Current data on the prevalence of COVID-19 both in rural areas and on rural public health practices are limited and may promote a false sense of security and insusceptibility regarding COVID-19 [12]. Poor safety practices in a community can compound and quickly overwhelm a community with limited resources to combat COVID-19.

In particular, Michigan found itself in the national spotlight as one of the first and hardest-hit states by COVID-19. There are no studies in Michigan that quantify knowledge or public health practices about COVID-19 in rural areas, nor studies exploring trends in misinformation and news sources in rural areas. This study investigates current knowledge and public health practices regarding COVID-19 in Gladwin, MI, the types of media and communications the community uses to keep updated on COVID-19 news and safety guidelines, trends between misinformation and specific news sources, as well as considerations for health communication efforts in combating COVID-19.

## Materials and methods

In our study, 258 patients completed a survey at a primary care health clinic in Gladwin, MI. All patients older than 18 years old that had an appointment during the recruitment period were eligible to participate.

Upon arrival for their scheduled appointment, patients were asked to participate in the survey before their provider visit. The completion of the survey indicated implied consent. All responses were anonymous. Survey responses were collected and recorded at the end of each day. Data collection occurred for two months between September 2020 and November 2020.

Statistical analysis was primarily conducted using SAS, version 9.4 (SAS Institute Inc., Cary, NC). Descriptive statistics provided were counted (percentage). Multivariable logistic regression was adopted to identify associations between demographic variables including COVID-19 news sources and response variables for the risk of transmission and spread of COVID-19. The penalized likelihood estimated by Firth [13] was proposed for the multivariable logistic regression with rare events. The 95% confidence interval for the odds ratio would be claimed as significant when the interval does not contain 1. Multiple linear regression was used to examine associations between demographic variables including COVID-19 news sources and response variables for the attitude toward COVID-19 and the vaccine. All analytical results were considered significant when p-values ≤ 0.05. With all the variance inflation factors being less than 10, the proposed models did not have multicollinearity issues.

## Results

Survey results

Table 1 reflects the responses of 258 participants over a two-month period. Most participants were over the age of 65 (n=81), majority Caucasian (n=224), and majority female (n=151). The highest level of education reported by most participants was high school (n=157).

**Table 1:** Descriptive statistics in count (percent) for categorical variables.

Variable	n (%)
Age	
18-24	19 (7.4%)
25-34	25 (9.8%)
35-44	45 (17.6%)
45-54	35 (13.7%)
55-64	51 (19.9%)
65+	81 (31.6%)
Race	
Caucasian	224 (90.3%)
Other	24 (9.7%)
Gender	
Male	83 (35.5%)
Education	
(Some) HS	157 (69.2%)
Bachelor	31 (13.7%)
Master+PhD	13 (5.7%)
Trade	25 (11.0%)
Risk Reduction Methods	
Handwashing	252 (98.4%)
Drying hands with hot air	40 (15.6%)
Treatment with hydroxychloroquine	23 (9.0%)
Transmission of COVID-19	
Through respiratory droplets when an infected person coughs, sneezes or talks.	240 (93.8%)
By touching a contaminated surface and then touching your eyes, nose, or mouth before washing your hands.	222 (86.7%)
Between people within about 6 feet of one another	155 (60.5%)
News Sources	
Television	139 (58.9%)
Website	86 (36.4%)
Social media	71 (30.1%)
Friends and family	59 (25.1%)

Participants demonstrated they were well informed about COVID-19 transmission and risk reduction, correctly reporting that handwashing (98.4%) and mask usage in public spaces (91.3%) were effective risk reduction methods and that the virus is spread through respiratory droplets when an infected person coughs, sneezes, or talks (93.8%). Participants were also asked about their agreement with statements about COVID-19 (1=strongly disagree; 5=strongly agree) and their likelihood of getting a COVID-19 vaccine (1=extremely unlikely, 4=extremely likely). On average, participants were more willing to get a COVID-19 vaccine than not (mean=2.58, SD=1.174). A majority agreed that it is possible to be infected with COVID-19 without any signs or symptoms (mean=4.52, SD=0.829). However, many participants also believe that COVID-19 is no more dangerous than other viruses such as the common flu (mean=2.84, SD=1.603). Most participants agreed that wearing a face mask can help prevent the spread of COVID-19 (mean=4.38, SD=1.035) and although most people agreed that social distancing is important to slow the spread of COVID-19 (mean=4.49, SD=0.955), only 60% of the participants believed that the virus is spread between people within about 6 feet of one another. On average, participants reported that they did not agree that they could trust their news sources (mean=2.73, SD=1.406).

The most common reasons reported by participants who stated they chose not to wear a face mask were that they did not feel wearing a mask helps to reduce transmission of COVID-19 (n=16), the mask is physically uncomfortable to wear (n=14), and the mask is a restriction on their freedoms (n=8). Also, over a quarter of participants (n=6) that reported that hydroxychloroquine is a COVID-19 risk reduction method (n=23) also reported getting their COVID-19 news and updates primarily from the CDC.

Misinformation trends across demographics

Overall, older participants demonstrated that they were better informed and reported higher adherence to safe public health practices such as mask usage and social distancing than younger participants. Participants over age 65 were more likely to agree that recommended public health practices such as wearing a face mask and social distancing help prevent the spread of COVID-19 than younger participants aged between 35 and 44 years old. Not only were older participants more likely to agree with recommended practices, but they are also four times more likely to actually wear a mask in public spaces (OR=0.18; CI [0.036, 0.931]) and more likely to get a vaccine than younger participants (Table 2).

**Table 2:** Multivariable logistics regression between age and knowledge of COVID-19 spread reduction methods: social distancing at six feet apart and wearing a mask in public spaces. Odds ratios (OR) and 95% confidence intervals are listed.

	Social distancing			Mask use		
	OR^2^	95% for OR		OR^2^	95% for OR	
Variables		Lower	Upper		Lower	Upper
Age 18-24 (ref=65+)	2.831	0.667	12.021	0.219	0.028	1.741
Age 25-34 (ref=65+)	1.808	0.564	5.799	0.283	0.045	1.782
Age 35-44 (ref=65+)	4.795	1.323	17.38	0.184	0.036	0.931
Age 45-54 (ref=65+)	5.66	1.708	18.751	0.363	0.064	2.048
Age 55-64 (ref=65+)	4.837	1.46	16.024	0.376	0.067	2.106

There were no associations with education level and misinformation found in this analysis. The data support that White participants are more likely to get a vaccine for COVID-19 than non-white participants (β=0.81; p=0.013). Male participants are more likely to get a vaccine for COVID-19 than female participants (β=0.55; p=0.003). Female participants are more likely to trust their news sources (β=-0.54 p=0.012), agree that wearing a face mask can help prevent the spread of COVID-19 (β=-0.33; p=0.040), and agree it is possible to be infected with COVID-19 without displaying any signs or symptoms than male participants (β=-0.34; p=0.018) (Table 3).

**Table 3:** Associations between agreement with COVID-19 statements and demographic and news source variables. Multiple linear regression analysis with Βeta (p-value). TV = Television; CBS5, CNN, FOX = Local television media sources; NS* = non-significant

Variables	Likelihood of getting a vaccine for COVID-19	Wearing a face mask that covers your nose, mouth and chin can help prevent the spread of COVID-19.	Social distancing is important to slow the spread of COVID-19	COVID-19 is no more dangerous than other viruses, such as the common flu.	It is possible to be infected with COVID-19 without displaying any signs or symptoms.	I know where to get reliable and updated information regarding COVID-19.	I can trust my news sources.
Age 25-34 (ref=65+)	-0.86 (0.005)	NS*	NS*	NS*	NS*	NS*	NS*
Age 35-44 (ref=65+)	-0.66 (0.015)	-0.71 (0.002)	-0.44 (0.045)	NS*	NS*	NS*	NS*
Age 55-64 (ref=65+)	-0.79 (0.003)	NS*	NS*	NS*	NS*	NS*	NS*
Ethnicity (ref=Non-White)	0.81 (0.013)	NS*	NS*	NS*	NS*	NS*	-0.70 (0.044)
Gender (ref=Female)	0.55 (0.003)	-0.33 (0.040)	NS*	NS*	-0.34 (0.018)	-0.46 (0.003)	-0.54 (0.012)
Website (ref=No)	NS*	NS*	NS*	NS*	0.32 (0.050)	NS*	NS*
Television (ref=No)	0.66 (0.002)	0.45 (0.015)	0.53 (0.002)	NS*	NS*	NS*	0.79 (0.002)
Newspaper (ref=No)	NS*	NS*	NS*	NS*	NS*	NS*	NS*
Friends/Family (ref=No)	NS*	NS*	-0.38 (0.015)	0.65 (0.017)	NS*	0.32 (0.048)	NS*
TV_CBS5 (ref=No)	NS*	NS*	NS*	NS*	NS*	NS*	-0.66 (0.024)
TV_CNN (ref=No)	NS*	NS*	NS*	NS*	NS*	NS*	0.85 (0.022)
TV_FOX (ref=No)	NS*	NS*	NS*	NS*	NS*	NS*	-1.18 (0.001)

Misinformation trends across news sources

Participants who reported family and friends as a primary new source were also more likely to agree that COVID-19 is no more dangerous than the common flu (β=0.65; p=0.017). Additionally, participants who get their news primarily from family and friends are less likely to agree that social distancing is important to slow the spread of COVID-19 (β=-0.38; p=0.015). The most reported new source was television (n=139). Television viewers were more likely to trust their news than participants who reported they did not get their news from television (β=0.79; p=0.002). CNN viewers reported that they could trust their news sources whereas CBS and FOX viewers were less likely to report that they could trust their news sources (Table 3). The breakdown of reported news sources by age can be found in Table 4. Overall, social media was reported as a primary news source in greater proportions by younger participants than older participants.

**Table 4:** Number of participants who reported social media, television, or friends and family as a primary news source by age group in count (percent). Percentages are calculated as (n/number of participants in age group).

		Primary news source reported		
Age	Number of participants in age group	Social Media	Television	Friends & Family
18-24	19	6 (31.6%)	7 (36.8%)	7 (36.8%)
25-34	25	11 (44.0%)	10 (40.0%)	6 (24.0%)
35-44	45	15 (33.3%)	16 (35.6%)	10 (22.2%)
45-54	35	7 (20.0%)	16 (45.7%)	7 (20.0%)
55-64	51	13 (25.5%)	29 (56.9%)	6 (11.8%)
65+	81	19 (23.5%)	63 (77.8%)	23 (28.4%)

## Discussion

Our study found that this sample of rural respondents was well informed about appropriate public health guidelines and virus transmission and suggest the current health communication practices have been effective in conveying COVID-19 public health messages. Most participants correctly report that handwashing (98.4%) and mask usage in public spaces (91.3%) were effective risk reduction methods and that the virus is spread through respiratory droplets when an infected person coughs, sneezes, or talks (93.8%). Although most people agreed that social distancing is important to slow the spread of COVID-19 (mean=4.49; SD=0.955), only 60% of the participants believed that COVID-19 spreads between people within about six feet of one another. This suggests that there may be dissonance between the term social distancing and its definition. 

Correlations between specific demographics and misinformation

White participants in this sample were more likely to get a vaccine for COVID-19 than non-White participants. However, due to the small number of non-White participants in this sample, more data and studies are needed to generalize this result to larger populations. This conclusion is supported by general sentiment in non-White and African American communities, in particular, that medical abuses as a result of medical racism have occurred, but more importantly, they are still occurring [14]. This is consistent with a 2021 study by Khubchandani et al. describing how COVID-19 vaccine hesitancy was highest in racial and ethnic minorities due to concerns regarding safety and efficacy or lack of health insurance or financial resources [15]. Widespread COVID-19 vaccine uptake and herd immunity will continue to be a challenge in the United States as the paradigm shifts from lacking supplies to administer the vaccine to a willingness to receive a vaccine.

Older participants demonstrated that they were better informed and reported higher adherence to safe public health practices such as mask usage and social distancing than their younger counterparts. This could explain the trend in young people engaging in unsafe practices such as “super spreader events” over the summer [16], perhaps due to preliminary communications of COVID-19 as a disease that did not significantly affect younger populations. The number of participants of this sample who participated in super spreader events was not collected in this study. 

Correlations between sources of news and misinformation

During the surge of the pandemic in April 2020, 67% of Americans were watching more news coverage, down to 36% in July 2020 [3]. Increased TV news consumption early in the COVID-19 pandemic may not have changed pre-existing ideas or understandings, as people likely choose to watch news channels that align with their existing belief system [17].

Additionally, social media has been a source of misinformation during the pandemic [7]. Because only 30% of participants reported using social media as a primary news source, this may explain why this sample was generally well-informed about COVID-19. Potentially, this may also be a reporting bias, as participants may not have wanted to claim social media as a primary news source. Instead, television was the most reported primary news source. Consistent with our findings, a 2020 study by Miller et al. describes how rural residents are more likely to use local television channels as a primary new source for both local and non-local stories [18].

We found that family and friends may be more trusted than other news sources for rural residents, thus community-based public health interventions through churches or schools, for example, may be more effective in rural communities. These findings are opposite those of a 2020 study by Wheldon et al. which found that 59.1% of rural respondents trusted family/friends as health information sources while a greater percentage (69.7%) of rural respondents trusted the government as a health information source [11]. Notably, the study also found trust in health information from government agencies only differed by rurality in bivariate chi-square analyses; however, after adjusting for covariates this association was no longer statistically significant.

The data suggest that interventions for COVID-19 in rural Michigan should focus on areas beyond education because participants are overall well-informed across demographics and between consumers who use different news sources. The idea of a knowledge-behavior gap is well described in the literature [19-22]. While current public health campaigns have increased general public knowledge about COVID-19, knowledge does not necessarily result in behavioral changes [23]. Other domains that may be contributing to this knowledge-behavior gap are self-efficacy [24], emotion [25], and political partisanship [26].

 A three-part approach to preventing COVID-19 was outlined by Ge et al., 2020: (1) identify the source effectively and control the infection; (2) break the transmission cycle; (3) protection of the vulnerable population [27]. In particular, (2) breaking the transmission cycle requires specific behaviors of members of society, including appropriately using hand sanitizers, wearing facial masks, and avoiding physical contact as much as possible. The translation of an understanding of information into public health behavior needs to be ensured.

The COM-B behavioral model has been adopted to analyze human behavior during the COVID-19 pandemic. The Capability to enact, the Opportunity to enable, and the Motivation to perform the behavior all should be present for the behavior to occur. The role of the government or another credible source of information must be identified in order for the public to trust policies, strategies, information, and guidance. The messenger needs to be deemed credible and the message needs to be relevant and achievable [28]. Therefore, interventions should also consider health behavior and promotion campaigns through local news outlets and community institutions for rural residents, as our data supports these as potentially credible news sources for this group. Furthermore, healthcare providers can be an important avenue for intervention, as the participants in our study were patients seeking healthcare and were overall well-informed about COVID-19. Future studies should investigate the role of providers as trusted sources by patients in reducing COVID-19 misinformation [29].

Limitations

Participants were recruited at a clinic, introducing inherent bias because patients in Michigan are required to wear a mask when they enter the building, thus only 8.7% of participants report never or rarely wearing a mask. The paper format of the survey resulted in many participants filling out the questionnaire incorrectly or incompletely. These data were unable to be captured. Lastly, the novel, widespread, and highly publicized nature of COVID-19 information in the news was difficult to capture in this research model and represents a unique challenge in this type of research [30].

## Conclusions

This study addresses the gap in the literature regarding accurate community knowledge and practices of COVID-19 public health guidelines and its relation to news sources and public trust in a rural area. In Gladwin, MI, misinformation, and mistrust exist in the community, but most patient participants demonstrate an understanding of public health guidelines and COVID-19. However, understanding public health guidelines did not necessarily result in expected behaviors. Our results suggest tailoring rural public health communication efforts to younger populations and utilizing both local television and community-based interventions to close this knowledge-behavior gap. Overall, these patients are well-informed, thus COVID-19 public health communications should focus on how to promote protective health behaviors beyond knowledge-only approaches.
